# 基于超高效液相色谱-电雾式检测的槐糖脂指纹图谱

**DOI:** 10.3724/SP.J.1123.2022.12025

**Published:** 2023-08-08

**Authors:** Qinling CAO, Xiaodan ZHAO, Guobin SHEN, Zhuqin WANG, Hongyang ZHANG, Min ZHANG, Ping HU

**Affiliations:** 1.华东理工大学化学与分子工程学院,上海市功能性材料化学重点实验室,上海 200237; 1. Shanghai Key Laboratory of Functional Materials Chemistry, School of Chemistry and Molecular Engineering, East China University of Science and Technology, Shanghai 200237, China; 2.赛默飞世尔科技(中国)有限公司,上海 201206; 2. Thermo Fisher Scientific (China) Co., Ltd., Shanghai 201206, China; 3.华东理工大学药学院,上海市细胞代谢光遗传学技术前沿科学研究基地,上海 200237; 3. Shanghai Frontiers Science Center of Optogenetic Techniques for Cell Metabolism, School of Pharmaceutical Sciences, East China University of Science and Technology, Shanghai 200237, China

**Keywords:** 超高效液相色谱, 电雾式检测器, 指纹图谱, ultra-high performance liquid chromatography (UHPLC), charged aerosol detector (CAD), fingerprint

## Abstract

槐糖脂具有良好的抗菌、抗病毒等生物学活性以及温和、低毒、环境友好的特点,是目前最具有市场前景的生物表面活性剂之一。但其组成成分复杂,尚缺乏有效的质量评价方法。针对槐糖脂无紫外吸收的特点,该研究选择了灵敏度高、重现性好、适用于无紫外吸收或弱紫外吸收物质检测的电雾式检测器(CAD),建立了槐糖脂的超高效液相色谱(UHPLC)指纹图谱。槐糖脂样品采用80%乙醇水溶液为提取溶剂,在液固比10∶1(mL/g)条件下超声提取10 min,利用Thermo Fisher Scientific Hypersil Gold色谱柱(150 mm×2.1 mm, 1.9 μm)进行分离,以乙腈-0.01%(v/v)甲酸水溶液作为流动相梯度洗脱,流速0.2 mL/min,柱温40 ℃,电雾式检测器检测,检测器参数设置为幂律函数1.0,采集频率5 Hz,过滤常数3.6,雾化温度45 ℃。此外,利用超高效液相色谱-四极杆飞行时间质谱(UHPLC-QTOF-MS)对槐糖脂指纹图谱中的色谱峰进行鉴定,共鉴定出16个化合物,包括8个酸型槐糖脂、6个内酯型槐糖脂和2个脂肪酸类化合物。方法精密度、重复性和24 h稳定性试验结果显示,15个特征峰相对于对照峰(油酸)的相对保留时间和相对峰面积的相对标准偏差(RSD)均小于3.0%(*n*=6)。采用上述方法对17批槐糖脂样品进行测定,相似度评价结果显示,17批样品的相似度数值均在0.965及以上,不同批次的槐糖脂样品之间化学成分差异较小,内在质量较为一致。该研究建立的方法稳定可靠,可用于槐糖脂的质量评价,为槐糖脂的生产工艺研究与开发利用奠定了基础。

## Abstract (en)

Sophorolipids are secondary metabolites produced during fermentation by nonpathogenic yeasts. These molecules are amphiphilic and consist of a hydrophilic sophora sugar moiety and a hydrophobic hydroxylated fatty acid. Based on their degree of esterification, sophorolipids can be divided into the acid and lactone types. Sophorolipids are highly promising biosurfactants with good antibacterial, antiviral, and other biological activities. Moreover, they are characterized by mildness, low toxicity, and environmental friendliness. However, their composition is quite complex, and effective methods for their quality evaluation are lacking. Since sophorolipids do not absorb ultraviolet (UV) light, common UV detectors are unsuitable for fingerprint establishment. In this study, we first selected a charged aerosol detector (CAD) to establish the ultra-high performance liquid chromatography (UHPLC) fingerprint of sophorolipids. The detector had high sensitivity, good reproducibility, and excellent suitability for the detection of substances with no or weak ultraviolet absorption. We then evaluated the similarities between 17 batches of sophorolipid samples. The samples were extracted by ultrasound for 10 min in 80% ethanol aqueous solution at a liquid-solid ratio of 10∶1 (mL/g) and then separated on a Thermo Fisher Scientific Hypersil Gold chromatographic column (150 mm×2.1 mm, 1.9 μm). Separation was performed using acetonitrile-0.01% (v/v) formic acid aqueous solution as the mobile phase via gradient elution. The flow rate was 0.2 mL/min, and the column temperature was 40 ℃. The CAD was used under the following conditions: power function of 1.0, data rate of 5 Hz, filter constant of 3.6, and evaporation temperature of 45 ℃. The chromatograms and retention times of the sophorolipids were compared, and 16 common peaks with strong responses, good resolutions, and stable retention times were selected as characteristic peaks. Oleic acid was chosen as the reference peak because it achieved good separation and a strong chromatographic response in all batches of samples. UHPLC-quadrupole time-of-flight mass spectrometry (UHPLC-QTOF-MS) was used to identify chromatographic peaks in the sophorolipid fingerprints. The results were combined with the retention time rule of the sophorolipids, leading to their identification based on matching with the results of the primary database, the precise relative molecular mass and fragmentation rule of secondary fragments, a self-built database, and the PubChem database. Sixteen compounds were identified, including eight acid sophorolipids, six lactone sophorolipids, and two aliphatic acids. The results of precision, repeatability, and 24 h stability tests indicated that the relative standard deviations (RSDs) of the retention times and peak areas of the 15 characteristic peaks relative to the control peak (oleic acid) were less than 3.0% (*n*=6). Seventeen batches of sophorolipid samples were analyzed, and the similarity values of all fingerprints were found to be 0.965 or higher. Little differences in chemical composition were observed among the different batches of sophorolipid samples, and the quality of the sophorolipids was relatively consistent. The fingerprint established in this study is stable and reliable; it can be used for the quality evaluation of sophorolipids and lays a solid foundation for future research on production technology and the development and utilization of sophorolipids. The successful application of a universal CAD to the fingerprint establishment of sophorolipids also provides a reliable solution for the fingerprint establishment of substances with no or weak ultraviolet absorption.

槐糖脂(sophorolipids)是由非致病性酵母菌发酵产生的次级代谢产物,其分子具有两亲性,由亲水性的槐糖部分和疏水性的羟基化脂肪酸部分构成,按内酯化程度分类,可分为酸型和内酯型两大类。槐糖脂的生物合成产量高^[1⇓-3]^,有良好的抗菌、抗病毒、抗肿瘤等生物学活性^[4⇓⇓-7]^,且具有温和、低毒、生物可降解和环境友好的特点,是最具有市场前景的生物表面活性剂之一,在化工、环境、食品、医药等领域有较高的研究和应用价值^[7⇓-9]^。槐糖脂的组成成分复杂,目前,在其物质基础和质量评价研究方面,槐糖脂指纹图谱研究暂未见报道。

电雾式检测器(charged aerosol detector, CAD)作为一种新型通用型检测器,具有灵敏度高、重现性好、线性范围宽等优势。且对非挥发性的化合物具有一致的响应,尤其适用于无紫外吸收或弱紫外吸收的物质检测^[10,11]^,广泛应用于天然产物、药物、表面活性剂等的分析检测^[12⇓⇓⇓-16]^,在中药指纹图谱研究中也表现出良好的应用潜力^[17⇓-19]^。

槐糖脂是无紫外吸收物质,难以利用常见的紫外检测器进行指纹图谱分析。本研究利用CAD检测器建立了槐糖脂的UHPLC-CAD指纹图谱,并利用超高效液相色谱-四极杆飞行时间质谱(UHPLC-QTOF-MS)对指纹图谱中的特征峰进行鉴定,以期为槐糖脂内在质量的整体控制提供思路,促进槐糖脂在更广领域的推广以及应用。

## 1 实验部分

### 1.1 仪器、试剂与材料

Ultimate 3000超高效液相色谱仪,配备Corona Veo RS CAD检测器(美国Thermo Fisher公司); Agilent 1290 UHPLC-6530 QTOF MS联用仪(美国Agilent公司); KQ-500DE型数控超声波清洗器(江苏昆山超声仪器有限公司); EPED-E2-10TF实验室级超纯水发生器(南京易普易达科技发展有限公司); PB1501-N精密天平(瑞士Mettler Toledo公司); CPA225D分析天平(德国Sartorius公司); 0.22 μm有机系尼龙针头式过滤器(日本Shimadzu公司)。

甲醇、乙腈(色谱纯,美国ACS公司);无水乙醇(分析纯,上海沃化化工有限公司);甲酸(分析纯,上海泰坦科技股份有限公司)。对照品油酸(批号O1008-1G,纯度≥99%)购自美国Sigma-Aldrich公司。17批槐糖脂样品(依次标记为S1~S17)均由实验室以蔗糖和菜籽油作为碳源,通过*Starmerella bombicola*菌株发酵得到。

### 1.2 实验方法

#### 1.2.1 对照品溶液制备

精密称取油酸对照品适量,加乙醇制成质量浓度为4 g/L的对照品溶液,摇匀,4 ℃下冷藏备用。

#### 1.2.2 供试品溶液制备

取槐糖脂样品约2.0 g,置具塞锥形瓶中,精密加入20 mL 80%乙醇水溶液,密塞,称定质量,超声(功率500 W,频率40 kHz)提取10 min,放冷,再称定质量,以80%乙醇水溶液补足失重,摇匀,过0.22 μm微孔滤膜,取续滤液,即得。

#### 1.2.3 UHPLC-CAD分析条件

采用Ultimate 3000超高效液相色谱仪,配备Corona Veo RS CAD检测器;Hypersil Gold色谱柱(150 mm×2.1 mm, 1.9 μm,赛默飞世尔科技中国有限公司)。流动相:乙腈(A)-0.01%甲酸水溶液(B);梯度洗脱:0~37 min, 40%A~60%A; 37~53 min, 60%A~90%A; 53~60 min, 90%A~100%A。柱温40 ℃;流速0.2 mL/min;进样量1 μL。幂律函数1.0,采集频率5 Hz,过滤常数3.6,雾化温度45 ℃,气速调节模式Analytical。

#### 1.2.4 UHPLC-QTOF-MS分析条件

采用Agilent 1290 UHPLC-6530 QTOF液相色谱-质谱联用仪,色谱条件同1.2.3节;电喷雾离子源(ESI),在负离子模式下扫描,采用全扫描模式(full MS)采集一级质谱数据,采用自动二级离子模式(Auto MS/MS)采集化合物的二级碎片数据。优化后的质谱参数如下:干燥气流量10 L/min;干燥气温度350 ℃;雾化气压力210 kPa(30 psi);毛细管电压3500 V; Skimmer电压65 V;八极杆射频电压750 V;毛细管出口电压150 V;碰撞能为30 eV;扫描范围为*m/z* 50~1600。

#### 1.2.5 指纹图谱测定

取17批槐糖脂样品制备供试品溶液,采用1.2.3节色谱方法进行分析,记录指纹图谱。

## 2 结果与讨论

### 2.1 提取方法优化

采用单因素考察法,取同一批样品(编号S1)制备供试品溶液,对提取溶剂比例、液固比(mL/g)和超声时间进行筛选,以指纹峰总峰面积作为评价提取效率的标准,确定最佳提取条件。在液固比为10∶1、超声时间为10 min时,考察不同提取溶剂(纯水、20%乙醇、40%乙醇、60%乙醇、80%乙醇、纯乙醇)的影响。在80%乙醇的条件下指纹峰的总峰面积达到最大值,遂确定提取溶剂中乙醇比例为80%。在提取溶剂为80%乙醇水溶液、超声时间为10 min时,考察不同液固比(5∶1、10∶1、15∶1、20∶1、25∶1)的影响。当液固比为10∶1时,供试品指纹图谱的总峰面积较液固比为5∶1时有明显提升,而当液固比继续增大时总峰面积趋于稳定,表明当液固比为10∶1时对样品的提取已趋近完全,故选定液固比为10∶1。在提取溶剂为80%乙醇水溶液、液固比为10∶1时,考察不同超声时间(0、10、20、30、40 min)的影响。超声提取10 min时总峰面积即达到稳定,超声时间延长总峰面积不再有显著增加,因此选定超声时间为10 min。最终确定提取方法为:80%乙醇水溶液作为提取溶剂,液固比10∶1,超声时间10 min。

### 2.2 色谱条件优化

本实验考察了Hypersil Gold (150 mm×2.1 mm, 1.9 μm)、Agilent Eclipse Plus C18 RRHD (150 mm×2.1 mm, 1.8 μm)、Agilent ZORBAX RRHD Eclipse Plus C18 (100 mm×3 mm, 1.8 μm)以及Phenomenex kinetex XB-C18 (150 mm×2.1 mm, 1.7 μm)色谱柱的分离效果,考虑仪器耐压与色谱柱的使用寿命,最终选择分离效果最佳、柱前压力较低的Hypersil Gold色谱柱(150 mm×2.1 mm, 1.9 μm)用于槐糖脂的指纹图谱分析。

考察了不同流动相(乙腈-水、乙腈-0.01%甲酸水溶液、乙腈-0.02%甲酸水溶液、乙腈-0.04%甲酸水溶液)、流速(0.20、0.25、0.30 mL/min)、柱温(35、40、45 ℃)对槐糖脂指纹图谱的影响,不同条件下的槐糖脂指纹图谱见图1。流动相中加入甲酸可抑制槐糖脂中大量存在的酸类物质的电离,获得良好的峰形;进一步考察甲酸含量,发现其体积分数为0.01%时即可达到良好的分离效果。流速为0.20 mL/min时,相较于较高流速,不存在色谱峰重叠、展宽的现象,分离效果良好。柱温为40 ℃时,相较于45 ℃, 50 min后色谱峰分离度更好;而与柱温35 ℃时相比,柱前压力更低,最终选择40 ℃为槐糖脂指纹图谱的分离温度。结果显示,以流动相乙腈-0.01%甲酸水溶液进行梯度洗脱、流速0.20 mL/min、柱温40 ℃时,各色谱峰峰形和分离效果良好,适用于槐糖脂指纹图谱分析。

**图1:**
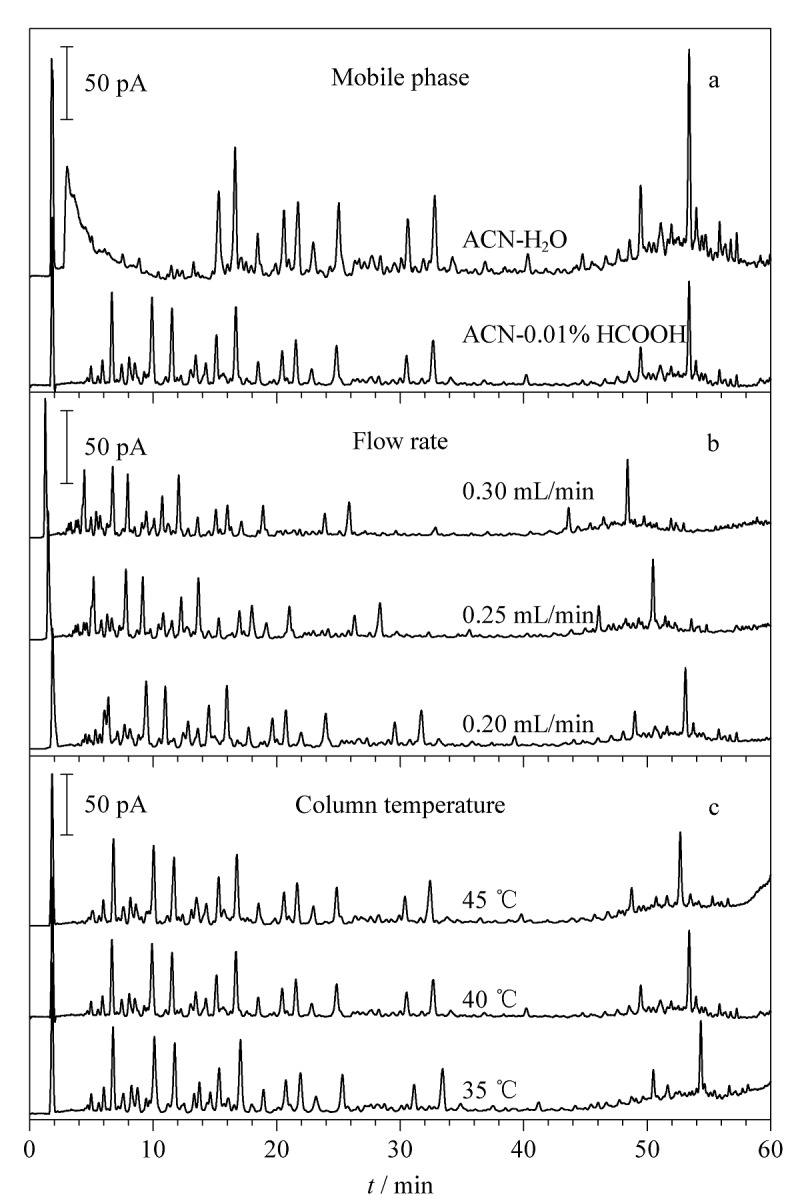
采用不同(a)流动相、(b)流速、(c)柱温时槐糖脂的UHPLC-CAD指纹图谱

对电雾式检测器的参数(幂律函数、采集频率、过滤常数、雾化温度、气速调节模式)进行了优化。确定了当CAD的参数设置为幂律函数1.0、采集频率5 Hz、过滤常数3.6、雾化温度45 ℃且气速调节模式选择Analytical模式时,指纹图谱分析可获得较高的信噪比。

### 2.3 指纹图谱的建立及特征峰的鉴定

将17批槐糖脂供试品溶液按1.2.3节中的色谱条件进行测定,具有代表性的槐糖脂样品(S1)的特征指纹图谱见图2。比较槐糖脂的色谱图及保留时间,选取响应较大、分离度良好,保留时间稳定的16个共有峰作为特征峰。其中,油酸(峰16)在各批次样品中均达到良好分离且具有较强的色谱响应,故将其作为参照峰(S)。

**图2:**
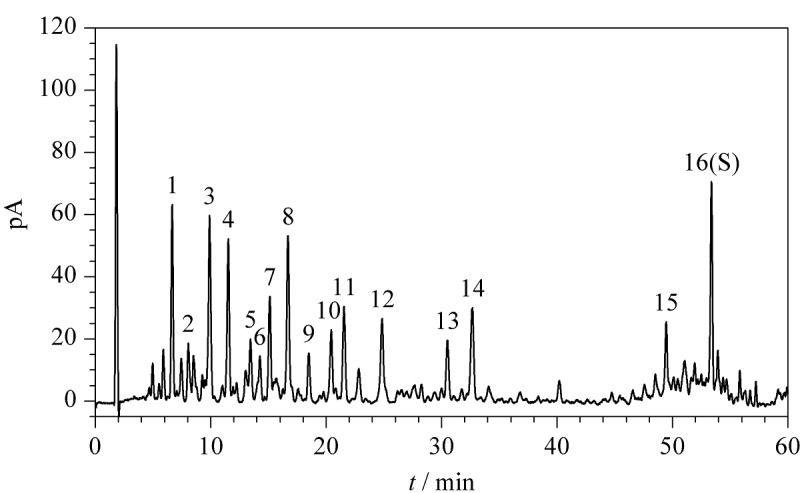
槐糖脂的UHPLC-CAD指纹图谱

采用1.2.4节中的色谱-质谱条件,利用UHPLC-QTOF-MS对槐糖脂指纹图谱的特征峰进行鉴定。槐糖脂产品通常是复杂的混合物体系,虽然不同槐糖脂分子的结构存在明显差异,但通常都具有通过*β*-1,2糖苷键连接的两个葡萄糖分子的亲水部分和一个由C16~C18羟基脂肪酸组成的疏水部分^[20]^。槐糖脂的内酯化和乙酰化使分子的疏水性增大,在色谱柱上的保留时间延长。羟基脂肪酸上羟基位置的差异也会导致保留时间的差异,羟基在脂肪酸的亚末端(*ω*-1)位置时保留时间较短,在末端(*ω*)位置时保留时间较长^[21]^,其中亚末端位置的羟基化在槐糖脂中更加常见^[22]^。

根据一级数据库匹配结果、二级碎片精确相对分子质量和裂解规律、自建数据库(基于槐糖脂的结构特征,利用Java语言在Eclipse集成开发环境下编写的槐糖脂理论分子式数据库)以及PubChem数据库,结合以上槐糖脂的保留时间规律,对指纹图谱中的16个特征峰进行鉴定,结果见表1。16个化合物中包括8个酸型槐糖脂、6个内酯型槐糖脂和2个脂肪酸类化合物,其结构式见图3。

**表1:** 槐糖脂中部分化学成分的初步鉴定

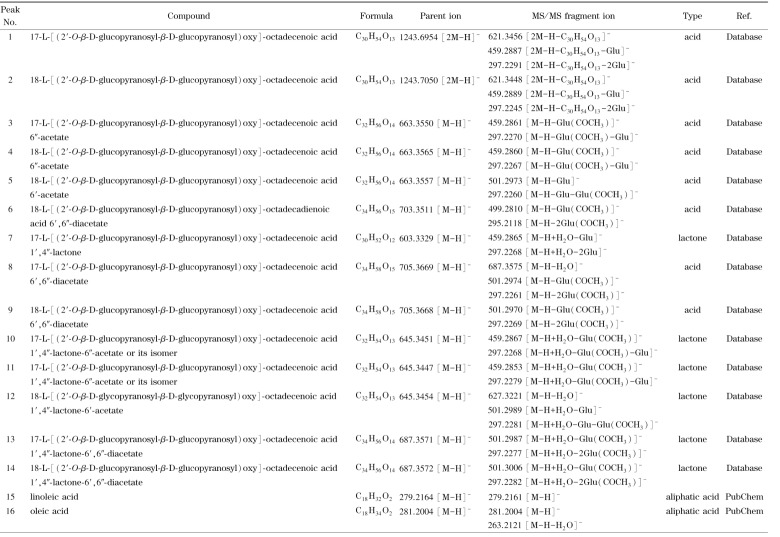

**图3:**
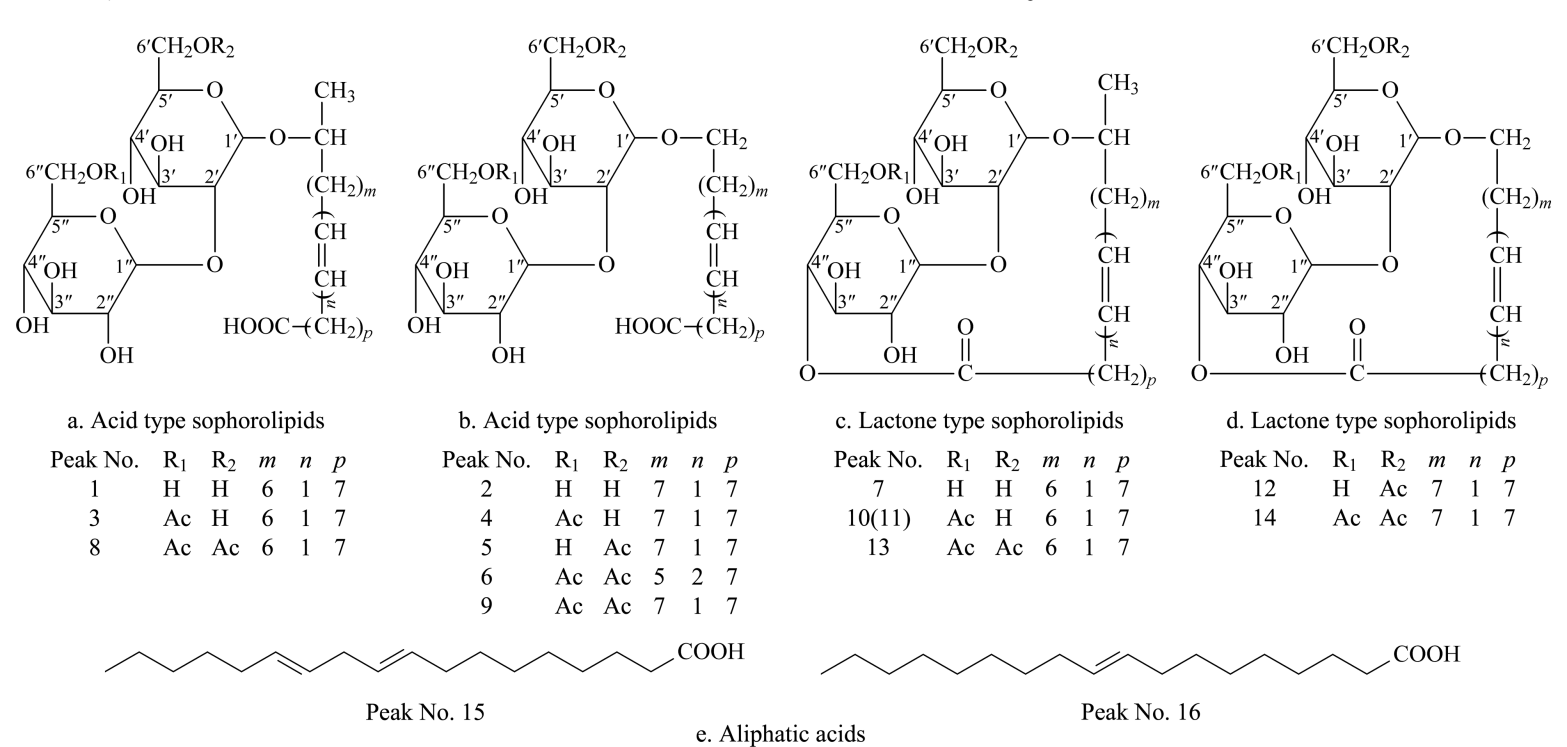
16个化合物的结构

### 2.4 方法学考察

#### 2.4.1 精密度试验

取同一批槐糖脂样品(S1)按1.2.2节方法制备供试品溶液,依照1.2.3节测定方法连续6次进样,记录所得色谱图,计算各特征峰相对于参照峰的相对保留时间和相对峰面积,结果见表2。测得各特征峰相对保留时间RSD≤0.15%,相对峰面积RSD≤2.95%,表明仪器精密度良好。

**表2:** 15个指纹峰的精密度、重复性和稳定性(*n*=6)

Peak No.	*t*_R_/min	Precisions			Repeatabilities			Stabilities			
		RSD_1_/%	RSD_2_/%		RSD_1_/%	RSD_2_/%		RSD_1_/%	RSD_2_/%		
1	6.499	0.05	1.10		0.15	1.16		0.30	0.73
2	7.907	0.10	2.48		0.13	1.54		0.29	2.32
3	9.729	0.05	1.04		0.19	2.10		0.26	2.98
4	11.417	0.05	1.18		0.15	0.61		0.18	1.29
5	13.415	0.10	1.37		0.21	1.28		0.14	0.93
6	14.222	0.08	2.95		0.12	2.01		0.11	0.96
7	15.107	0.12	2.05		0.19	1.88		0.12	1.23
8	16.665	0.15	1.90		0.10	1.37		0.19	1.81
9	18.472	0.10	2.17		0.09	2.74		0.16	2.31
10	20.428	0.10	2.04		0.07	2.60		0.16	2.80
11	21.536	0.04	0.94		0.05	1.00		0.12	0.87
12	24.869	0.05	1.90		0.10	2.62		0.10	1.32
13	30.513	0.04	1.85		0.05	2.05		0.07	1.82
14	32.657	0.04	1.38		0.04	1.05		0.06	1.57
15	49.458	0.01	1.93		0.02	1.27		0.02	2.63
16(S)	53.390	-	-		-	-		-	-

RSD_1_: RSD of relative retention time; RSD_2_: RSD of relative peak area.

#### 2.4.2 重复性试验

按1.2.2节方法平行制备槐糖脂(S1)供试品溶液共6份,依照1.2.3节测定方法分别进样分析,记录所得色谱图,计算各特征峰相对于参照峰的相对保留时间和相对峰面积,结果见表2。测得各特征峰的相对保留时间RSD≤0.21%,相对峰面积RSD≤2.74%,表明方法重复性良好。

#### 2.4.3 稳定性试验

取槐糖脂样品(S1)按1.2.2节方法制备1份供试品溶液,依照1.2.3节测定方法分别于0、2、4、6、12、24 h进样,记录所得色谱图,计算各特征峰相对于参照峰的相对保留时间和相对峰面积,结果见表2。测得各特征峰相对保留时间RSD≤0.30%,相对峰面积RSD≤2.98%,表明供试品溶液在24 h内稳定性良好。

### 2.5 相似度评价

将所有批次槐糖脂的色谱数据导入国家药典委员会“中药色谱指纹图谱相似度评价系统”(2012版),经中位数法多点校正之后,对色谱峰进行自动匹配,生成指纹图谱共有模式作为对照指纹图谱,进行相似度计算。17批槐糖脂样品的UHPLC-CAD指纹图谱及所生成的对照指纹图谱见图4,相似度结果见表3。数据显示,不同批次槐糖脂样品指纹图谱的相似度很高,其范围为0.965~0.995,表明本实验选取的不同批次槐糖脂样品之间化学物质群差异较小,内在质量较为一致,制备工艺合理,稳定性较好。

**图4:**
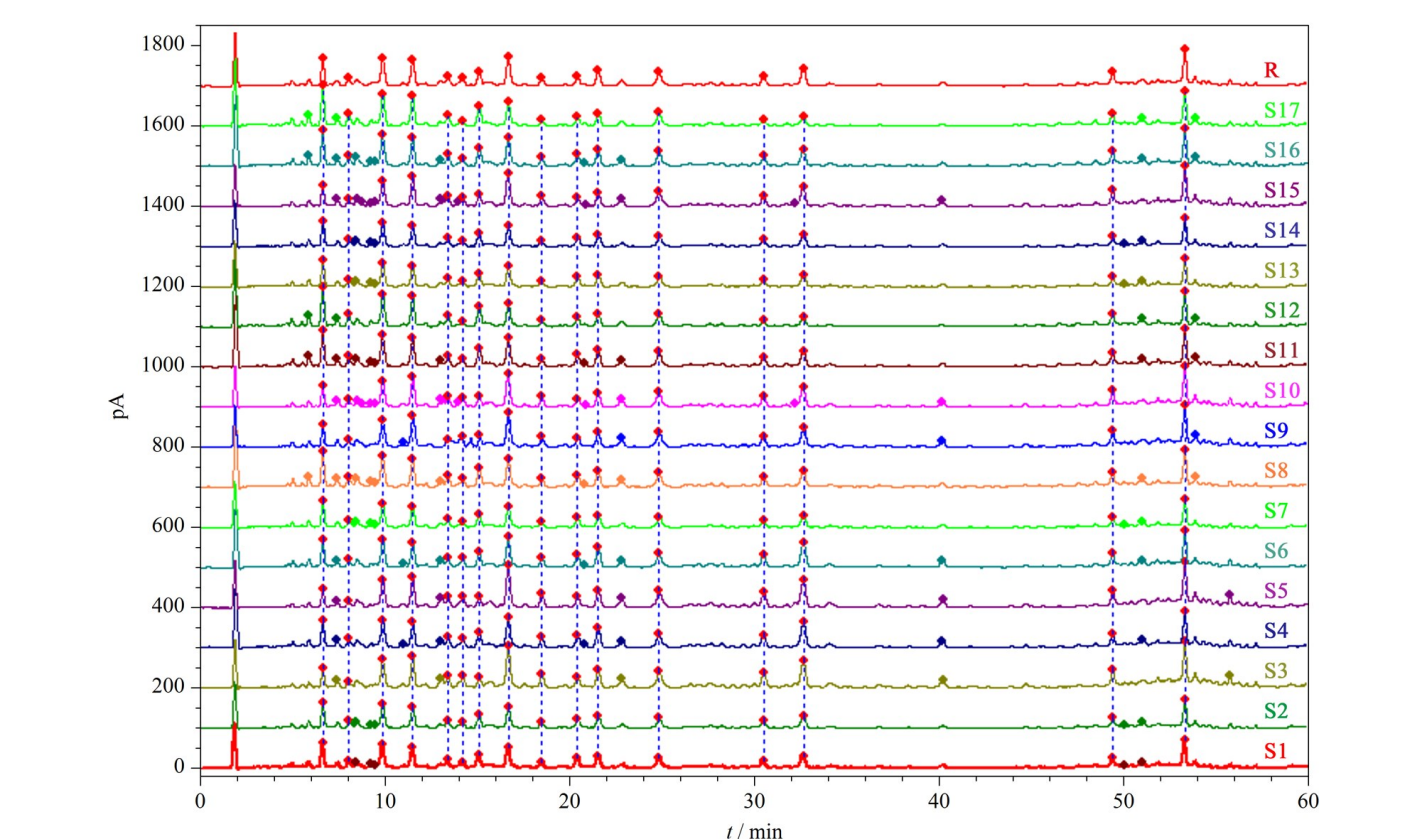
17批槐糖脂样品(S1~S17)的UHPLC-CAD指纹图谱及对照指纹图谱(R)

**表3:** 17批槐糖脂样品指纹图谱的相似度

Sample	Similarity	Sample	Similarity
S1	0.991	S10	0.989
S2	0.993	S11	0.993
S3	0.968	S12	0.965
S4	0.988	S13	0.991
S5	0.968	S14	0.992
S6	0.986	S15	0.989
S7	0.995	S16	0.993
S8	0.993	S17	0.965
S9	0.986		

## 3 结论

本研究建立了槐糖脂的UHPLC指纹图谱,并利用UHPLC-QTOF-MS对指纹图谱中的特征峰进行了鉴定,从整体性出发,为槐糖脂的质量评价提供了参考,以期促进槐糖脂的生产工艺、质量控制与开发利用研究。与此同时,通用型检测器CAD在槐糖脂指纹图谱建立中的成功应用,为无紫外吸收或弱紫外吸收物质的指纹图谱建立提供了可靠的解决办法。
